# Exploring dental professionals' perceptions of resilience to dental environment stress: a qualitative study

**DOI:** 10.1038/s41415-024-7953-z

**Published:** 2025-03-28

**Authors:** Marina Harris, Kenneth Eaton

**Affiliations:** 227684639283126090230 Associate Professor of Dental Education and Wellbeing, School of Dental, Health and Care Professions, Faculty of Science, University of Portsmouth, Portsmouth, United Kingdom; 300980229603577938683 Visiting Professor, School of Dental, Health and Care Professions, Faculty of Science, University of Portsmouth, Portsmouth, United Kingdom

## Abstract

**Aims** This study aimed to investigate what constitutes the meaning of resilience to dental environment stress for different members of the dental team, and how resilience is operationalised by these team members. It also aimed to clarify if there were different or similar sources of stress for different groups.

**Methods** Participants were recruited from dentists, dental therapists, dental hygienists, dental nurses, dental technicians and clinical dental technicians. In total, 22 participants provided informed consent to take part and were interviewed - 17 online and five in person. The interviews were audio-recorded and transcribed. The transcripts were member checked and then analysed. Codes were generated and grouped into themes. The University of Portsmouth Science and Health Faculty Ethics Committee approved the study.

**Results** Three themes with 11 sub-themes were identified. The sub-themes were: expectations associated with working environment; pressures associated with working environment; team dynamics; taking a balanced view; engaging with support networks; achieving work/life balance; diversity and variety in role; organisation; sense of purpose; gratitude; and positive relationships. Many of the stresses which were identified were common to all team members.

**Conclusions** The findings highlighted disparities and similarities with experiences which need to be addressed to further enhance dental professionals' wellbeing. They will also inform a planned national quantitative study.

## Key points

- Provides initial insights into dental team members' perceptions of how to be resilient to dental environment stress.
- Indicates that many causes of dental environment stress are common to all dental team members.
- Will guide investigators with regard to which questions to ask in a planned national follow-up study.

## Introduction

The World Health Organisation has defined mental health as ‘a state of wellbeing in which a person recognises one's full potential, copes with life's stresses, works fruitfully and contributes to society'.^1^ However, despite recent breakthroughs in the research on ‘resilience', its definition remains obscure. Particularly within the stressful field of dentistry, there is the need for more understanding of what resilience actually means to people, what factors enhance or challenge their resilience, and what strategies they employ to develop resilience throughout their careers. It is hypothesised that resilient dental professionals will be less of a risk to patient safety, and to themselves.

A study carried out among United Kingdom (UK) dentists found that 44% of the dentists who responded to the survey reported that their level of stress was exceeding their ability to cope with everyday requirements of practice.^2^ Likewise, a study which examined other members of the dental team found that mental health problems transferred to general practice, with 45% of dental therapists based in South West England reporting lower life satisfaction, worthwhileness, and happiness compared to the general public.^3^ These recent studies highlight how knowledge of preventive and protective resilience strategies is of paramount importance to improve wellbeing for dental professionals in order to reduce risk to personal and patient safety.

Resilience is commonly defined as ‘bouncing back' through positive adaptation in a context of significant adversity.^4^ Evolving research approaches resilience as a process existing on a continuum instead of making a binary distinction between the existence and non-existence of it as a trait, and it has been suggested that the level of resilience fluctuates due to environment, interpersonal interactions and personal resources.^4^^,^^5^ These include emotional intelligence, autonomy, cognitive flexibility, self-determination and self-worth. For example, in a study which investigated resilience within dental undergraduate education, dentists who were also educators felt that the academic setting enhanced their resilience, while the intimidating environment of general dental practices took over their ability to deal with challenges.^6^ The study also showed that dentists who perceived themselves as resilient dealt with the stress intuitively, whereas less resilient dentists indicated deliberate strategies, such as mindfulness, physical exercise and speaking out, to boost resilience.^6^

In aviation safety, it is recognised that in the case of an emergency, passengers are instructed to put on their own oxygen mask before helping others. This rationale applies equally to dental care professionals who need to look after their own safety to ensure that they can protect the safety of patients.

There is no universal conceptualisation of resilience as it depends on the context in which the term is used.^7^ Indeed, this lack of shared understanding of resilience within the field of dentistry has impeded the efforts to design or deliver resilience-enhancing education and strategies that are appropriate and meaningful to all members of the dental team.^8^

It appeared that no previous study had investigated the similarities and differences of types of stressors and coping mechanisms which are experienced by different members of the dental team in everyday practice and throughout their careers. Thus, the target population for the study included representatives from all registrant members of the dental team.

## Aims

This study aimed to investigate the factors which may erode or enhance resilience to dental environment stress for different members of the dental team, and how resilience is operationalised by these team members. It also aimed to clarify if there were different or similar sources of stress for different groups.

## Methods

Ethical approval was gained from the University of Portsmouth Science and Health Faculty Ethics Committee (SHFEC 2023 - 098). Participants were advised verbally and in writing that all information they provided was confidential and that their data would be anonymised. The sample size and composition consisted of participants from all seven groups of general practice registrants and specialist practice (Table 1). This provided a total sample size of 22 which reflected dental professionals from different regions and working environments. The sample comprised 10 male and 12 female participants.

**Table 1:** The sample size and composition of participants

**Registrant category**	Dentist x 5
	Dental nurse x 4
	Dental therapist x 2
	Dental hygienist x 4
	Dental technician x 2
	Clinical dental technician x 2
	Orthodontic therapist x 1
	Specialist x 2
**Geographical location**	Northern Ireland
	Scotland
	Wales
	England
	Channel Islands
**Systems working within**	National Health System
	Private
	Community Services
	Corporate
	Academia
**Career stage**	Very early
	Mid-career
	Late-career

Participants were a purposeful sample recruited via the authors' wide network of contacts throughout the profession. Very few participants were known directly to the researcher conducting the interviews, as most participants were recruited through network colleagues informing professionals within their own networks to contact the research team. The sample size was deliberately calculated to produce a manageable number of participants for the study, while reflecting the number of participants for each registrant category as relative to the lesser or greater numbers for that particular category on the General Dental Council (GDC) register. Using an interview guide of four, key, open questions, which was piloted and adapted in light of feedback (Table 2), 17 of the interviews were conducted via the use of the online Zoom platform. The remaining five interviews were conducted opportunistically face-to-face with delegates who were attending a dental conference. The interviews were conducted by the lead author, also a GDC registrant. Consequently, through the process of reflexivity, the researcher sought to maintain neutral position throughout. Written and verbal consent was obtained for all 22 semi-structured interviews which were audio-recorded, professionally transcribed and thematic analyses carried out. Due to the nature of the topic, all participants were emailed links to support networks, should they need them, after each interview. The interviews were conducted over the period November 2023 to March 2024.

**Table 2:** The interview guide

Demographics	What is your role in the dental team? How long have you been qualified? Where is your geographical location of work? Do you work in private/NHS/academia? What gender do you identify with?
Interview questions	What are the key factors which cause you stress within your professional role? How have you dealt with stressful situations relating to your professional role previously? Can you describe any strategies which you have subsequently implemented to prevent some of the stressors you have mentioned occurring in the first place? What is it about your role which brings you contentment?

Interview transcriptions were sent to the participants, who were asked to confirm their accuracy before the analysis was carried out. Thematic analysis of all of the data were undertaken using Braun and Clarke's (2006)^9^ six phases of thematic analysis.

Initial codes were generated from across the entire data set and then collated into potential themes. These themes were then reviewed and further defined and named. All of the data were analysed independently by the two authors who are experienced in qualitative methodology, and three themes encompassing eleven sub-themes were identified.

## Results

The 11 themes which were identified are listed in Table 3. Key points within each theme will now be reported.

**Table 3:** The eleven themes which were identified

**Themes**	**Sub-themes**
Theme 1: factors challenging resilience	Expectations associated with working environment Pressures associated with working environment Team dynamics
Theme 2: factors enhancing resilience	Taking a balanced view Engaging with support networks Achieving work/life balance Diversity and variety in role Organisation
Theme 3: positive emotions enhancing resilience	Sense of purpose Gratitude Positive relationships

### Theme 1a: expectations associated with the working environment

It was not surprising that most of the participants mentioned patient expectations as a major factor which challenged their resilience. This was more apparent from participants who worked within the NHS system:

- ‘*Maintaining that patient expectation, while fulfilling your NHS contractual obligation is difficult'* (P1).

Another participant reflected on how NHS dentistry is wrongly perceived by some people, and said ‘*a lot of the older generation seem to think everything should be free and certain things should be available to them that maybe were in the past, but now aren't'* (P13). However, patient expectations were also an issue for those working in the private referral sector. For one participant:

- ‘*The challenge is, it's very hard to be able to say that we're not able to help people, because of the expectation that we should be treating all of the patients'* (P17).

### Theme 1b: pressures associated with the working environment

Time management, financial concerns and workload were the prominent themes which emerged from the data. Time pressure was a challenge for participants from both the clinical practice setting and the dental laboratory setting alike. Participants who worked in practice stated pressures which arose from their own patient schedule: *‘I would say I think it's a high-pressured environment, and often cases we'll have patients that are back-to-back 30 minutes from 9am in the morning all the way through ‘til seven o'clock in the evening, so time is the real restriction here”* (P9), as well as time pressures for those who supported the clinicians*: ‘knowing that you're going to run late at the end of the day pretty much every day and it's really hard to just get everything done, it's a whirlwind, isn't it, from morning to afternoon'* (P14). From the dental laboratory perspective, time pressures seemed to emerge from unrealistic expectations from dentists regarding turnaround times for laboratory work. One participant stated *‘you know, we can't always turn things round in two days, we're not waiting for their work to come in, we've got clients from all round the county coming in'* (P21), while another elaborated on how the lack of dialogue could sometimes be detrimental for patient satisfaction:

- ‘*You've got a time thing, you've got to get it back on time but no one will get it. So, you get this huge stress level. Does the lab start with it even though they don't feel safe to get on with it? Or do they get into trouble for saying, well, we've been trying to get you to speak to us about it for ages, we haven't done it yet? So, it's a no-win situation'* (P19).

As expected, practice owners cited financial pressure as a source of stress:

- ‘*Trying to run a dental practice with the best equipment and the best materials and provide a real top-notch service, but also still being really fair to patients in terms of costings and making it affordable for people can be quite difficult'* (P14).

However, financial pressure was also a concern from other members of the team*:*

- *‘Often there is an external pressure to sort of up sale on certain services to meet certain quotas, that I'm sure the management here at the practice are under a bit of pressure and stress to achieve. So, from my experience, that's something that's pushed onto the clinicians'* (P9).

Workload was mentioned more by participants who were in management positions, with particular reference to paperwork, which would often need to be taken home to complete. However, workload was quite a strong theme from those participants who were also working in academia. A participant who worked in practice and academia stated:

- ‘*In general dental practice, one saving grace is once you are out of the clinic, you are out of the clinic, you don't need to work. However, in academia, you can carry on with work outside your eight to five remit and that is the increased workload and maintaining that work/life balance. Basically, working all weekend has now become more and more of a common thing I do*' (P1).

This was similar to another academic:

- ‘*You can't switch off because you've got Microsoft Teams on your phone, a student messages you on a Saturday night, like what can I do, I'm meant to be off, it's switching off sometimes is the challenge*' (P21).

Within this same theme, there was also mention of certain levels of anxiety within the working environment. This was generally in relation to feeling out of one's comfort zone. However, for one of the newly qualified participants, dealing with dissatisfied patients was a worry:

- ‘*I think to some degree I perhaps wasn't equipped to deal with patients that were either distressed or unhappy for whatever reason, and so that was a challenge*'.

It was also a source of anxiety as to how well their competence was perceived by the dentist:

- ‘*Because we don't want to come across as incompetent in front of the dentists. We don't want the dentist to stop referring you the patient, because you asked them some things. I don't want to give them the […] projecting like “oh she's always asking things and she doesn't know what she's doing”'* (P10).

### Theme 1c: team dynamics

This theme affirmed how vital good team work and communication was for all the participants in the study. Some participants spoke about a hierarchal system, which for some, was an obstacle to improving their working conditions, as well as instilling a reluctance to approach management with a concern. Participant 7 stated:

- ‘*There's a hierarchy and you have to be really strong to push to get best treatment care and best conditions. I don't like the hierarchy'* (P7).

This was echoed by another participant who similarly stated:

- *‘I wouldn't necessarily say that that person hasn't got resilience because they don't want to address their manager. You know what I mean? I think it's more they're just fearful of the hierarchy system in the practice*' (P11).

Lack of communication was a repetitive theme which was elicited mainly from the data from the dental technician (DT) and clinical DT participants. Participants spoke about the feeling of ‘disconnect' and the lack of understanding of roles:

- ‘*I personally don't feel we cross over enough during our training and that's the downside with technicians being trained in separate environments to dentists and other dental care professionals. And I think that's a lacking part and I think that's where we need to try and build that education because it'll help the relationships going forward'* (P21).

They also discussed how lack of regular feedback from the dentist furthered their feeling of isolation and stated that feedback was usually only given if there was a problem with the lab work. For one laboratory owner, feedback was particularly important:

- ‘*Because there's no clinic nearby, you're sending stuff by post, and these jobs are going in and out each day, dental clinicians don't get feedback. So, we introduced that “how did I do” card, so like a little postcard thing, that they can put in, we encourage them to put […] but actually that is, you know, that is such a simple thing, but that could be so valuable'* (P20).

Lack of autonomy was also an element of team dynamics which challenged resilience. This was predominantly reported by the team members who supported the clinicians:

- ‘*I think with dental nursing, you don't really have any real autonomy. So, your day is not yours to create or to map out*' (P15).

However, it was also an issue for clinicians who worked in the corporate setting:

- ‘*Because if you were straight out of university, you probably would say thank you for helping me run my book. But I've been running my book perfectly successfully and fulfilling my targets without their interference and I couldn't see why they should have that level of interference over me'* (P4).

### Theme 2a: taking a balanced view

The data strongly indicated that the study participants had the ability to put things into perspective. Positive mindset and self-acceptance were constant elements in many of the interview transcripts. Admitting mistakes was not an issue for this participant:

- ‘*So, yes, you will make mistakes, yes, there will be really stressful times, there will be times where you've got to put your hand up and say, I made the wrong call, and there's nothing wrong with that'* (P2).

In acceptance of their individual role and responsibility, one participant stated:

- ‘*But in your head, you think, well, that's my role because who else is going to do it? I've got no other team members to do it and the business needs to continue and it needs to be managed today, and that's what I need to do*' (P15).

Acceptance of level of control over certain situations was also reflected in the interviews:

- ‘*I do try to leave it at home a lot more because, you know, I used to do things like, I'd worry if a patient was okay after an extraction, you'd ring them in the evening and actually when you boil it down, you can't do anything until the next day anyway, there's no point'* (P5).

In recognising the bigger picture, one participant stated:

- ‘*And we all think we want to be supported by the practice owners, but they too have their own challenges as well. So sometimes we're forgetting we need to see them as humans. So, if you try to make their life a little bit easier, maybe you will get something that you want'* (P8).

For one of the newly qualified participants, optimism for sustainability of the career ahead was apparent:

- ‘*I think with increasing experience, the likelihood is there'll be more positive experiences in which you can reflect on, and it almost reinforces your place within the dental team'* (P9).

The need for a paradigm shifts in mindset to enhance resilience was very clear from one participant:

- ‘*And there is a bit of a general kind of negative feeling from labs towards clinics. We change that tone, that's not the case, dentists are our friends, practice managers are our friends. We communicate, we speak to each other, and we encourage good feedback, well, we encourage feedback whether it's good or bad, because we need to learn'* (P20).

### Theme 2b: engaging with supportive networks

This was a theme which every single participant mentioned to some extent or other. Some participants discussed how important having a mentor was to their ability to thrive within the profession:

- *‘In the workplace, I think you need someone there, whether it's someone in your practice, someone you can go to afterwards, and that, you know […] I suppose, you can take it wider, outside of life, but in dentistry, I think it's really important'* (P2).

Furthermore, how important a mentor was at the start of one's career:

- *‘I think it is for people like [name] who is really resilient, I think he's amazing, and he is such a great mentor for other dentists, younger dentists. That's really inspired me and given me real hope for my career forward*' (P3).

Mentor support was also valuable for one participant's advancing career:

- ‘*Useful early on in my specialist career was to go to sit with another specialist and watch how they […] basically how they ran a consultation, how they interacted. To watch somebody who had a very well-polished act'* (P18).

Participants also engaged with peer support networks: *‘you can feel very isolated because I've got no-one to turn to. I've got no dentists to turn to if I need one. So, that is very isolating. So, I can, at any time, email my colleagues and ask them how they've dealt with this problem because we mostly have the same problems*' (P16); with outside support networks: *‘I find that while we have a sort of open-door policy between practice managers and principal dentists as well and I'm fully aware that that's an open door, rather than go there, I find that I'm more likely to go to people outside of the practice, whether that be friends, family, partner. I think outside of that as well'* (P9); and finally, the importance of family support: ‘*and I bounce ideas off my wife and get things into context a little bit and then, ah, actually it be alright, calm down and take the emotion out of it, then, yeah, it's actually not that bad'* (P20). However, participants did not necessarily require solutions to their problems:

- ‘*It's being able to get it off your chest, not necessarily get it sorted but just relieve the pressure, if you like, by vocalising, by verbalising things'* (P6).

### Theme 2c: achieving work/life balance

This was mainly perceived by those participants who had the ability to ‘switch off' and not allow work to follow them home:

- ‘*I'm quite fortunate in that my work doesn't really follow me home, so to speak. So, it's nice that whatever happens here while I'm working tends to stay here. It's not a burden that follows me once I finish'* (P9).

For another participant, working within private practice made a difference:

- ‘*Working as a dental nurse in private practice I just find an absolute dream, it's very structured, it's very easy. As soon as you walk out of that room, out of the surgery, to me you're finished, that's it done, and yeah, I don't have to take that home with me at all'* (P14).

Within the field of academia, not allowing digital technology to encroach on home life was emphasised by this participant:

- ‘*I think the other action is to take the applications off your phone or turn the notifications off, that's what I do, at the end of the day I use the function on my iPhone to turn off notifications for particular applications so I don't get them coming through'* (P21).

Also, within academia, having the set holiday structure compensated for the long hours that were worked in term-time:

- ‘*When we have the summer break, the Christmas break and stuff, the break away from students and most of all, happiness*' (P1).

Unsurprisingly, participants who had a reduced working week perceived themselves as having a good work/life balance. However, these were generally participants who were in later stages of their career, and had the financial stability to make that choice.

### Theme 2d: diversity and variety in role

This showed how some participants used variety and diversity in their everyday practice to maintain enthusiasm for their work. Some participants actively pursued education and training for personal and professional development: *‘from day one I've been doing the training and each qualification leads on to the next qualification. So, each time I'm going through that I'm thinking to myself, right, okay, where am I going next? What do I want to do? And the whole process has led me to where I am now'* (P15), while others immersed themselves in activities that served the industry as a whole: ‘*I immerse myself in our profession. I immerse myself in our industry, So I've immersed myself in the wider world of dentistry, it doesn't scare me anymore, I know most of it very well. So, because I've got that very wide range, I see over those stresses now'* (P19).

### Theme 2e: organisation

This was a key element for enhancing resilience for most of the participants. Indeed, for some of the dental nurse participants, working outside of their paid hours so that they could have an organised day was the norm. For the clinicians, it was apparent how important having autonomy was to how much control they had over how their day was organised. This was important for their feelings of being able to provide the best treatment for their patients. For two participants, this seemed to come with experience:

- ‘*I'm brilliant at time management and I'm a bit like, it's taken me years to get, you know, the patients aren't getting a disservice, put it like that, do you know, because I've had to go on loads of courses, really up skill'* (P7)
- ‘*Because I don't think it makes a healthy work environment if you're stressed from a time point of view. So, I've worked hard to both organise the way in which the staff and my nurses work*' (P12).

Flexibility and insight into own interpersonal skills were also deemed important for another*:*

- ‘*So, I think the only way I can reduce my anxiety for tomorrow is to be as prepared as I can but also as flexible as I can. I also always rely on the fact that I'm reasonably laidback as an individual and approachable'* (P18).

### Theme 3a: sense of purpose

All participants described how their role in the dental team fulfilled their sense of purpose and they perceived their work ‘made a difference' to the lives of others. These were strong sentiments which the more experienced participants described as the reasons why they remained in the profession. One participant stated:

- ‘*Then when you walk down the street in a small community they will say, you did this for my nan, you did this for my granddad, you did this for […] so, it gives you a great satisfaction and that is where that side of things isn't about the money'* (P22).

A positively functioning team was also deemed important to this participant*:*

- ‘*My clinicians, my dentists get really excited about cases that they're working on, so they're real sharers and it's really positive to see how excited and how their patients start and how they finish, it makes you feel good actually, knowing that you've contributed to that in whichever way it is'* (P14).

The perception of making a difference was also very meaningful when working in unique circumstances:

- ‘*But I know that I can make people feel better, in their last hours particularly. And it's amazing what a little bit of mouth care can do to someone's wellbeing'* (P16).

### Theme 3b: gratitude

This showed that although participants were cognisant of the stressful nature of the profession, on the whole, they generally felt contentment and a sense of ‘privilege' that their role afforded them:

- ‘*We're all very lucky, especially people here, we pretty much love what we do. We've all got friends who don't do jobs that they love every day'* (P2).

Being grateful for having a skillset that could be used for the greater good was also reflected on*:*

- ‘*I volunteer for Dentaid which I find quite therapeutic, actually, in terms of there's always someone worse off than you are but you don't always recognise that or see it, and when you do see it, you actually realise that you're very privileged, really'* (P4).

Gratitude was more bluntly stated by this participant:

- ‘*You know, we're indoors, we're listening to the radio, we're quite comfortable. Yes, patients can be difficult, situations can be difficult, but in the grand scheme of things, we're not in a warzone, we're not down a coal mine*' (P13).

### Theme 3c: positive relationships

Working within an environment which facilitated complete trust from members of the public brought contentment at work among the participants. Participant 4 stated very much the essence which was echoed by the majority of participants:

- ‘*Well, I think it does because you meet people and they tell you things and it has always surprised me what things people tell you about their personal life that you don't need to know but they just volunteer the information. They've got this thing that they're worried about and they tell you about it and you're a listening pair of ears and I think that's good for them*' (P4).

Good relationships with other members of the dental team were also a key feature:

- ‘*I think I'd always make the point we used to hold study groups and we used to go and talk to our clients and go and visit them regularly, and the ones that I knew more personally generally had a much better relationship and, on the whole, generally found that helped*' (P21).

## Discussion

Resilience is a multi-factorial phenomenon which acts on a continuum. It is far more complex than just ‘bouncing back' from adversity.^4^^,^^10^ Indeed, factors which enhance resilience are thoughts and actions which are both ‘preventive' and ‘reactionary', and these lived experiences of preventive and reactionary measures to dental environment stressors were shared by the study participants.

Delivering safe and effective patient care is contingent on dental professionals' ability to overcome the many challenges which working in an intense environment entail.^10^ The current study explored participants' perceptions of resilience to dental environment stress associated with their role in the dental team. Among the individual team members, there were many similar factors which either challenged or enhanced their resilience to the stressors they faced. However, there were also differences to these challenges and enhancers which only pertained to specific groups of registrants.

Participants in the study discussed very similar aspects of environmental stressors which is well-documented throughout the literature.^8^ They reiterated the issues related to working within the restrictions of the National Health System (NHS), which were primarily stressors associated with time pressures and patient expectations. However, even for participants at the ‘top of the field' in private practice, patient expectations were also a source of stress to them. Patient-led stressors have been a constant theme within the literature over the last two decades,^11^^,^^12^^,^^13^ with fear of litigation reported as an emerging source of stress within dentistry.^12^ Interestingly though, this was not a theme which emerged from participants in this study. This is an encouraging finding in relation to participants' perceptions that they perceived themselves as working safely and effectively. It was not expectations from patients which were the source of stress for DTs, but unrealistic expectations from other members of the team. The lack of insight into the skill and expertise of the DTs, along with poor communication, seemed to be the major contributing factors for DTs sometimes feeling a lack of belongingness in the dental team. Previous studies have investigated dentists' knowledge of the role of other members of the dental team,^14^^,^^15^ with a more recent study reporting encouraging findings of improved knowledge from dentist colleagues regarding the expertise of other team members.^16^ The participants in this study concluded that better integration and collaboration at dental undergraduate level could be a solution to this issue, and so this should be implemented.

Unlike medical practice, the practice of dentistry is a business and relies on revenue obtained from payment of services. There was an uneasiness from one participant about feeling compelled to advocate treatment which they did not feel was necessary. This may be a dilemma for some groups of registrants who are not in a position to find work elsewhere. In its investigation into the public and the profession's understanding of ‘professionalism', the GDC found that the tension between patient interest and financial interests could indeed affect how treatment is provided in some cases.^17^

The UK's higher education is going through a financial crisis.^18^ Lack of recruitment of both national and international students has left many universities with a large deficit in their predicted budget. This has resulted in a freezing of staff recruitment across the sector. The stressors experienced by study participants who were in academia reflected this changing landscape. They described excessive workloads which encroached on their leisure time, as well as unrealistic student expectations of when they should be available. This is a worry for dental undergraduate education, as it may well be that academics will leave the sector due to burnout,^19^ but moreover, for the education of our future workforce, experienced dental professionals may not even wish to enter academia at all.

Anxiety is worrying about the effects that a past stressor had, or what a future stressor may have, on an individual.^20^ A low level of anxiety can be viewed as adaptive, and in many cases is a facilitator for resilience. For example, one participant spoke about reducing their anxiety about presenting a study day in an unfamiliar environment by having ‘back-ups' for worst-case scenarios. On the other hand, for one of the newly qualified participants, reluctance to seek advice and mentorship from the dentist for fear of being viewed as incompetent showed how their high level of anxiety was not adaptive. This ‘avoidance-coping' mechanism^21^ may have lessened the opportunity to learn and grow from an experienced colleague as opposed to only reaching out to their peers.

Autonomy refers to the desire for one's own behaviour to be intrinsically motivated, as opposed to coming from external sources, and is thought to be an expression of the self.^22^ In an autonomy-supporting environment, individuals tend to experience stressful situations as a challenge rather than a threat and cope with them successfully.^4^ Lack of autonomy over how their day was planned and their ‘perceived position' within the hierarchy of the dental team was echoed by all of the dental nurse participants. It was also a high source of stress for the dentists working in the corporate setting, as generally they had been used to having full control, and now found themselves being ‘told' what they could and could not do. On the other hand, good organisation was strongly linked to participants' perceptions of having autonomy. Moreover, control over one's day was a key component which participants felt improved their resilience. Described as the individual who has the qualities of self-determination, independence and regulation of behaviour from within,^23^ a sense of autonomy reflects the tendency for individuals to evaluate themselves by their own standards and resist social pressures. Therefore, it was unsurprising that findings from those participants who had autonomy to be organised felt able to eradicate most of the dental environment stressors, whereas those without autonomy, could not.

One of the ‘enablers' associated with the ability to ‘get over' worrying about mistakes or things that were outside their control was participants' ability to exercise self-compassion. This is an ability to accept failure as the human condition and not over-identify with one-off negative experiences so that one can move forward.^24^ In studies which have examined self-compassion in healthcare, they found that self-compassionate physicians reported greater work engagement, less emotional and physical exhaustion, and greater satisfaction with professional life.^25^ Moreover, the literature supports the notion that those individuals who have self-compassion are more likely to be compassionate towards other people.^24^^,^^25^^,^^26^^,^^27^ Tolerance of others' inadequacies is a quality that is of critical importance to both the provision of patient care and working as a dental team.

Supportive networks as a tool for resilience is a major contributor to our coping mechanism. Social support is also a two-way process and studies have shown that in stressful circumstances, the giving of social support can be as effective to an individual as receiving it.^28^ Moreover, it is the type and quality of the relationship to the support provider (e.g. friend versus authoritarian figure) which has been shown to have an impact on the stress-buffering effect of the support.^29^ The participants used the networks which are identified in the literature as being beneficial and were very similar for all participants. However, there was a reluctance from participants to use ‘in-house' support and preferred other avenues. This also aligns with the literature which suggests that self-efficacy and self-worth can be undermined if the social support which has been given is perceived as having been given disingenuously.^30^ This is an important observation which may suggest a training and development need for those practices whose support schemes are not used by the very people for whom they are there to serve.

Throughout a lifetime, it is inevitable that to reach certain goals, individuals will prioritise activities in the service of certain values, while curtailing or omitting others.^31^ Designating one valued direction as more important than another can be helpful when it is a temporary measure. However, if the temporary imbalance becomes more permanent, it can result in a narrowing of the behavioural repertoire and a reduction in the quality of life.^32^ To counteract the stressor of work overload, participants used various strategies to accommodate an improved work/life balance. Participants shared ideas of how they made positive actions to enable them to ‘switch off'. For some, the aftermath of the COVID pandemic made them evaluate the importance of family and leisure time and subsequently reduced their working week.

It was apparent from all the participants with experience that whatever their role was in the dental team, it brought aspects of ‘meaningfulness', which they perceived as mitigators to the stressors. These participants interpreted their experiences in terms of a meaningful life story, such as work which gave them a sense of purpose. At a biological level, purpose in life has been shown to predict both lower levels of allostatic overload and better emotional recovery from negative stimuli, and therefore increase resiliency to stress and depression.^33^ At a psychological level, it has been shown to be a strong predictor of an individual's perception of autonomy, and likewise may also increase resiliency to stress and depression.^34^ The participants also believed that their work made a difference. This sense of efficacy, even if it is an illusion, allowed individuals to interpret events in ways that supported the belief that they can make a difference in some important way.^35^ Privilege and a sense of gratitude were other elements of positive emotions which enhanced resilience. Emerging research on the beneficial impact of possessing an attitude of gratitude is gaining momentum, with studies showing positive effects on wellbeing in healthcare workers.^36^^,^^37^

Working within the field of dentistry requires a person-centred approach. Indeed, the experiences of positive relationships described by participants in the current study perfectly align with the renowned psychology theorists' descriptions of the fully functioning person as one that is able to show a basic trustworthiness of human nature, and the ability to respond to other individuals^38^^,^^39^ and when operationalised, is a beneficial coping strategy to stress.

### Limitations

While the results are valid for answering the research question, they should be interpreted with respect to limitations. Although the study carried out one-to-one interviews to provide personalised responses, it is quite likely that it is only participants who perceived themselves as resilient who volunteered to take part. Furthermore, the participant recruitment process was contingent on the authors' network contacts and so the net for participants may not have been cast as widely as it could have been. To overcome the fact that it was only a small team of two conducting the study, both authors exercised reflexivity and thought carefully and critically about how these influenced the research process, including interpretation of data.

## Conclusions

The study has provided further understanding of how resilience to dental environment stressors is operationalised in the everyday work experiences of the dental team. Moreover, the authors believe it is the first study to purposefully include representation from all registrants of the dental team. Although there were many similar experiences among the dental team, the findings also highlighted disparities with experiences which need to be addressed to further enhance dental professionals' wellbeing. On the other hand, despite the disparities among participants in this study, they were all able to discuss attitudes towards their work which portrayed the characteristics of being positively functioning individuals.

Learning about coping or preventive strategies that dental team members are using on a day-to-day basis to get through their working day has a fundamental influence on how we can interpret personal wellbeing and the safety of patients. This philosophy underpins recent initiatives for mental health frameworks within the profession both nationally and globally, and should be applauded.^40^^,^^41^ Such knowledge can only be seen as huge benefit to the profession as a whole, and needs to be disseminated to inform stakeholders in multiple arenas who have an interest in the wellbeing of a group of healthcare providers who make a vital contribution to the oral and holistic health of our population.

This study has identified a range of issues which the participants perceived as influencing their resilience and mental health. However, they were a very small group and may not necessarily reflect these issues for each group of dental professionals or nationally. There is a need for a national survey as a follow-up, with its questions informed by the results of the current study, with particular emphasis on questions exploring attributes of positive emotions which enhance resilience. There is also scope for more detailed qualitative studies to explore resilience within the specific groups of the dental profession which have previously been poorly presented within research.

## Data Availability

Data are available on request to the lead author.

## References

[1] World Health Organization. Mental health: strengthening our response. 2022. Available at https://www.who.int/news-room/fact-sheets/detail/mental-health-strengthening-our-response (accessed on May 2024).

[2] Collin V, O'Selmo E, Whitehead P. Stress, psychological distress, burnout and perfectionism in UK dental students. *Br Dent J* 2020; **229:** 605-614. 10.1038/s41415-020-2281-4 33188344

[3] Hallett G, Witton R, Mills I. A survey of mental wellbeing and stress among dental therapists and hygienists in South West England. *Br Dent J* 2022; DOI: 10.1038/s41415-022-5357-5. 10.1038/s41415-022-5357-5 PMC9746560 36513756

[4] Rolin H, Fossion P, Kotsou I, Leys C. Perspectives on resilience: trait or aptitude? *Rev Med Brux* 2018; **39:** 22-28. 10.30637/2018.17-050 29528595

[5] Mansfield C F, Beltman S, Broadley T, Weatherby-Fell N. Building resilience in teacher education: an evidenced informed framework. *Teach Teach Educ* 2016; **54:** 77-87.

[6] Fulton C, Carr A, Penlington C. Exploring the term ‘resilience' as understood and experienced by dental educators. *Eur J Dent Ed* 2020; **25:** 573-581. 10.1111/eje.12634 33220103

[7] Aburn G, Gott M, Hoare K. What is resilience? An integrative review of the empirical literature. *J Adv Nurs* 2016; **72:** 980-1000. 10.1111/jan.12888 26748456

[8] General Dental Council. Mental health and wellbeing in dentistry: a rapid evidence assessment. 2021. Available at https://www.gdc-uk.org/docs/default-source/research/mental-health-and-wellbeing-in-dentistry27973e06-eb0f-4ee2-b92f-7fee3d2baf5b.pdf (accessed May 2024).

[9] Braun V, Clarke,V. Using thematic analysis in psychology. *Qual Res Psychol* 2006; **3:** 77-101.

[10] Joyce S, Shand F, Tighe J, Laurent S J, Bryant R A, Harvey S B. Road to resilience: a systematic review and meta-analysis of resilience training programmes and interventions. *BMJ Open* 2018; DOI: 10.1136/bmjopen-2017-017858. 10.1136/bmjopen-2017-017858 PMC6009510 29903782

[11] Chipchase S Y, Chapman H R, Bretherton R. A study to explore if dentists' anxiety affects their clinical decision-making. *Br Dent J* 2017; **222:** 277-290. 10.1038/sj.bdj.2017.173 28232686

[12] Collin V, Toon M, O'Selmo E, Reynolds L, Whitehead P. A survey of stress, burnout and well-being in UK dentists. *Br Dent J* 2019; **226:** 40-49. 10.1038/sj.bdj.2019.6 30631165

[13] Myers H L, Myers L B. ‘It's difficult being a dentist': stress and health in the general dental practitioner. *Br Dent J* 2004; **197:** 89-93. 10.1038/sj.bdj.4811476 15272347

[14] Ross M K, Turner S. Direct access in the UK. what do dentists really think? *Br Dent J* 2015; **218:** 641-647. 10.1038/sj.bdj.2015.504 26068161

[15] Turner S, Tripathee S, MacGillivray S. Direct access to DCPs: what are the potential risks and benefits? *Br Dent J* 2013; **215:** 577-582. 10.1038/sj.bdj.2013.1145 24309791

[16] McClune L, Harris M, Reed D. A mixed-method study to establish the current level, nature, and attitude to the provision of treatment under direct access for dental hygienists and dental therapists who are members of the British Society of Dental Hygiene and Therapy. *Ann Clin J Dent Health* 2023; **12:** 5-12.

[17] General Dental Council. Professionalism: a mixed-methods research study. 2020. Available at https://www.gdc-uk.org/docs/default-source/research/professionalism---a-mixed-methods-research-study (accessed June 2024).

[18] Office for Students. Increased pressure on higher education finances. 2024. Available at https://www.officeforstudents.org.uk/news-blog-and-events/press-and-media/increased-pressure-on-higher-education-finances/ (accessed June 2024).

[19] Watts J, Robertson N. Burnout in university teaching staff: a systematic literature review. *Educ Res* 2011; **53:** 33-50.

[20] Dugas M J, Freeston M H, Ladouceur R, Rhéaume J, Provencher M, Boisvert J M. Worry themes in primary GAD, secondary GAD, and other anxiety disorders. *J Anxiety Disord* 1998; **12:** 253-261. 10.1016/s0887-6185(98)00013-9 9653683

[21] Kennerley H. *Managing anxiety: a training manual*. Oxford: Oxford University Press; 1990.

[22] Weinstein N, Ryan R M. A self-determination theory approach to understanding stress incursion and responses. *Stress Health* 2011; **27:** 4-17.

[23] Ryff C D. Happiness is everything, or is it? Explorations on the meaning of psychological well-being. *J Pers Soc Psychol* 1989; **57:** 1069-1081.

[24] Neff K. *Self-compassion: stop beating yourself up and leave security behind*. New York: Harper Collins, 2011.

[25] Babenko O, Mosewich A D, Lee A, Koppula S. Association of physicians' self-compassion with work engagement, exhaustion, and professional life satisfaction. *Med Sci (Basel)* 2019; **7:** 29. 10.3390/medsci7020029 PMC6409678 30759845

[26] Neff K D. The development and validation of a scale to measure self-compassion. *Self Identity* 2003; **2:** 223-250.

[27] Zessin U, Dickhäuser O, Garbade S. The relationship between self-compassion and well-being: a meta-analysis. *Appl Psychol Health Well Being* 2015; **7:** 340-364. 10.1111/aphw.12051 26311196

[28] Taylor S E. *The tending instinct: how nurturing is essential to how we live*. New York: Holt, 2002.

[29] Bland H W, Melton B F, Welle P, Bigham L. Stress tolerance: new challenges for millennial college students. *Coll Stud J* 2012*;* **46:** 362-375.

[30] Rook K S. Social networks in later life weighing positive and negative effects on health and well-being. *Curr Dir Psychol Sci* 2015; **24:** 45-51. 10.1177/0963721414551364 PMC4564251 26366047

[31] Dahl J C, Plumb J C, Stewart I, Lundgren T. *The art and science of valuing in psychotherapy: helping clients discover, explore, and commit to valued action using acceptance and commitment therapy*. Oakland: New Harbinger, 2009.

[32] Lee C, Powers J R. Number of social roles, health, and well-being in three generations of Australian women. *Int J Behav Med* 2002; **9:** 195-215. 10.1207/s15327558ijbm0903_03 12360837

[33] Schaefer S M, Morozink Boylan J *et al*. Purpose in life predicts better emotional recovery from negative stimuli. *PLos ONE* 2013*;* DOI: 10.1371/journal.pone.0080329. 10.1371/journal.pone.0080329 PMC3827458 24236176

[34] Zilioli S, Slatcher R B, Ong A D, Gruenewald T L. Purpose in life predicts allostatic load ten years later. *J Psychosom Res* 2015; **79:** 451-457. 10.1016/j.jpsychores.2015.09.013 PMC4684637 26526322

[35] Baumeister R F, Vohs K D, Aaker J L, Garbinsky E N. Some key differences between a happy life and a meaningful life. *J Posit Psychol* 2013; **8:** 505-516.

[36] Cheng S-T, Tsui P K, Lam J H M. Improving mental health in health care practitioners: randomized controlled trial of a gratitude intervention. *J Consult Clin Psych* 2015; **83:** 177-186. 10.1037/a0037895 25222798

[37] Calleja P, Knight-Davidson P, McVicar A, Laker C, Yu S, Roszak-Burton L. Gratitude interventions to improve wellbeing and resilience of graduate nurses transitioning to practice: a scoping review. *Int J Nurs Stud Advan* 2024; **6:** 100188. 10.1016/j.ijnsa.2024.100188 PMC11080336 38746819

[38] Rogers C R. *On becoming a person: a therapist's view of psychotherapy*. London: Constable, 1967.

[39] Maslow A H. *Toward a psychology of being.* 2nd ed. New York: Van Nostrand, 1968.

[40] FDI World Dental Federation. Mental health and well-being for oral health professionals and dental students. Available at https://www.fdiworlddental.org/mental-health-and-well-being-oral-health-professionals-and-dental-students (accessed August 2024).

[41] British Dental Journal. New mental health wellness initiative launched. *Br Dent J* 2021; **230:** 632-633.

